# Molecular Characterization of Infectious Laryngotracheitis Virus from Broiler Diseased Chickens in Northern Vietnam During 2023–2025

**DOI:** 10.3390/ijms27177544

**Published:** 2026-08-23

**Authors:** Giang Thi Huong Tran, Vuong Trong Tran, Amonpun Rattanasrisomporn, Hieu Van Dong, Autchara Kayan, Jatuporn Rattanasrisomporn

**Affiliations:** 1 Faculty of Veterinary Medicine, Vietnam National University of Agriculture, Hanoi 131000, Vietnam; tthgiang@vnua.edu.vn (G.T.H.T.); dvhieuvet@vnua.edu.vn (H.V.D.); 2 Center for Advanced Studies for Agriculture and Food, Kasetsart University Institute for Advanced Studies, Kasetsart University, Bangkok 10900, Thailand; 3 National Center for Veterinary Diagnosis No. 2, Department of Animal Health and Production, Ministry of Agriculture and Environment, Hanoi 131000, Vietnam; vuong89.vet@gmail.com; 4 Interdisciplinary of Genetic Engineering and Bioinformatics, Graduate School, Kasetsart University, Bangkok 10900, Thailand; fgraapr@ku.ac.th; 5 Department of Animal Science, Faculty of Agriculture, Kasetsart University, Bangkok 10900, Thailand; 6 Department of Companion Animal Clinical Sciences, Faculty of Veterinary Medicine, Kasetsart University, Bangkok 10900, Thailand

**Keywords:** genetic characterization, infectious laryngotracheitis virus, PCR, phylogenetic analysis, Vietnam

## Abstract

PCR-based surveillance identified the infectious laryngotracheitis virus (ILTV) genome in 8 out of 14 (57.14%) commercial broiler farms across three Northern Vietnam provinces. Partial sequencing of the infected cell protein 4 (*ICP4*) and glycoprotein B, G, and J (*gB*, *gG*, and *gJ*) genes from the ILTV isolates obtained during 2023–2025 indicated that eight Vietnamese ILTV-positive samples were field ILTV strains. Additionally, high nucleotide identity was observed among the isolates, with ranges of 99.74–100% for the *gB*, 99.63–100% for the *gJ*, 99.66–100% for the *gG*, and 99.34–100% for the *ICP4* genes. Phylogenetic analyses based on four genes (*gB*, *gJ*, *gG*, and *ICP4*) revealed that the eight ILTV strains belonged to two distinct lineages, except for the *gJ* gene. Analysis of the *ICP4* gene showed that the five ILTV strains (VNUA-ILTV-02, VNUA-ILTV-05 to VNUA-ILTV-08) clustered with the United States Laryno Vac, Poulvac-ILT, and ON-2462242/1987 strains, whereas the remaining strains (VNUA-ILTV-01, VNUA-ILTV-03, and VNUA-ILTV-04) were in a distinct group. Furthermore, several amino acid changes were observed in the gB, gG, and gJ proteins of the characterized ILTV strains.

## 1. Introduction

The poultry industry is a key pillar of the agricultural economy in Vietnam, contributing notably to national food security through the supply of poultry meat and eggs as major sources of animal protein. Infectious laryngotracheitis (ILT) is a highly transmissible respiratory disease of chickens and represents a major cause of economic loss in the global poultry sector. The causative agent, infectious laryngotracheitis virus (ILTV), also known as Gallid herpesvirus 1, is classified within the genus *Iltovirus*, subfamily Alphaherpesvirinae of the family Herpesviridae [1].

The ILTV genome consists of a linear double-stranded DNA molecule about 153 kilobases in size. Its genome includes a unique long (UL) region and a unique short (US) region, both flanked by inverted repeat sequences (IRSs) and terminal repeat sequences (TRSs) [2]. The viral genome is predicted to encode about 80 protein-coding open reading frames (ORFs). Molecular typing based on PCR amplification and sequencing of the infected cell protein 4 (*ICP4*) gene remains the most widely used method for strain differentiation [3,4,5].

Chickens constitute the principal natural host of ILTV, with more severe and predominant clinical signs exhibited typically by mature birds [6]. In severe infection, clinical presentation includes marked respiratory distress, manifested by gasping, coughing, and tracheal exudate, which may progress to fatal airway obstruction [7,8]. Disease outcomes vary according to host immune status and viral virulence, with flock mortality potentially reaching 100% and case fatality rates exceeding 50% [9]. ILTV infection leads to substantial production losses, including increased chicken losses and reduced egg output in both commercial broiler and layer production systems [10].

In Vietnam, scientific reports are scarce regarding the molecular epidemiology of ILTV [11]. This knowledge gap necessitates targeted investigation of ILTV associated with current outbreaks in commercial broiler flocks and the performance of molecular characterization of circulating strains. Such findings are critical for elucidating viral diversity for optimizing control strategies against ILT within the country.

## 2. Results

### 2.1. ILTV Genome Detection Using PCR

**Figure 1:**
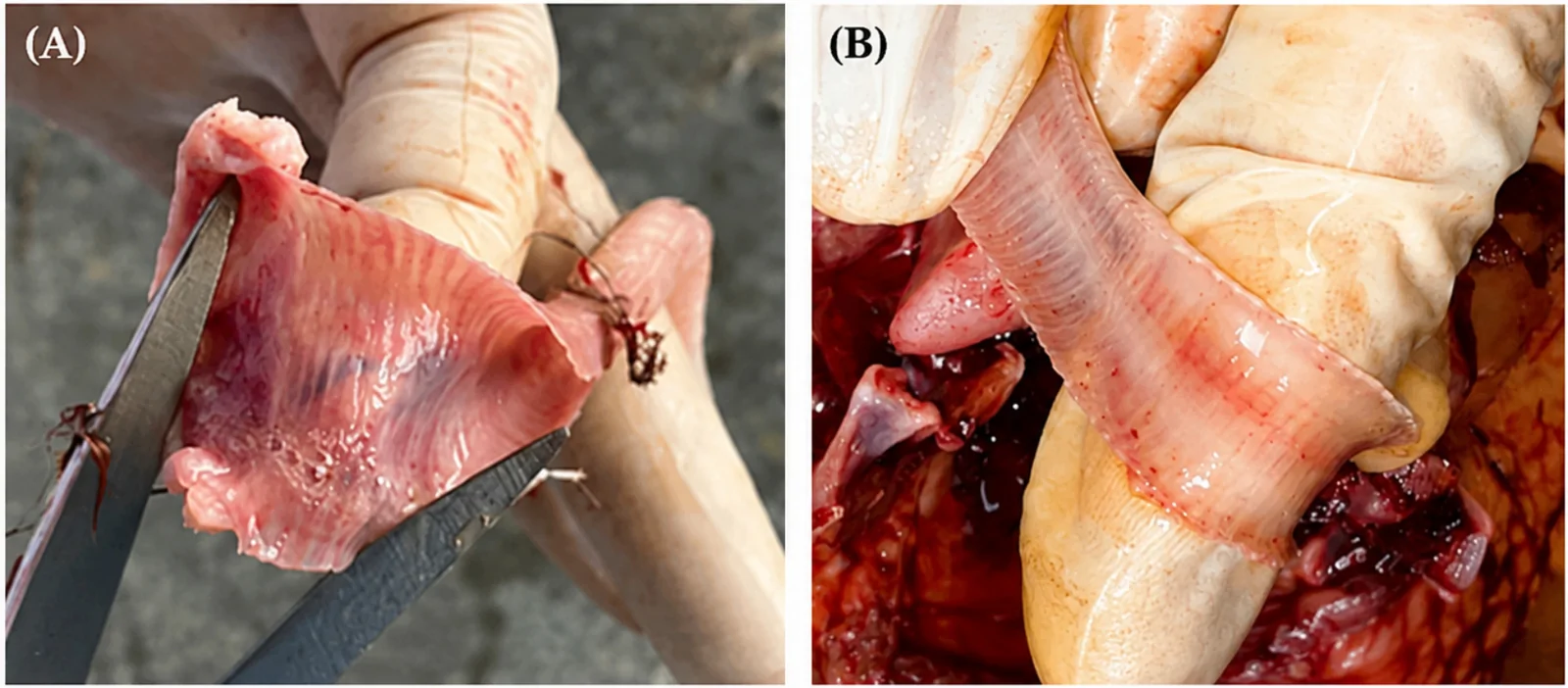
Post-mortem lesions of chickens with suspected ILTV infection: (**A**) Accumulation of mucus within the respiratory tract and (**B**) Hemorrhagic lesion of the tracheal mucosa.

The present study investigated flocks of predominantly broiler chickens containing 500–6000 individuals. Clinical signs observed among the sampled chickens included depression, poor growth performance, coughing, and gasping, with a mortality rate of 5–10%. Notably, frequent, excessive accumulation of mucus within the respiratory tract was observed, with some chickens exhibiting hemorrhagic lesions of the tracheal mucosa (Figure 1).

The present study indicated that ILTV DNA was detected by PCR in 8 of 14 (57.14%) diseased ILTV-suspected broiler chicken farms in the three northern cities/province. More specifically, three samples from BN, one sample from TN, and four samples from HN were determined to be ILTV genome-positive. The ILTV strains were Vietnam/VNUA/ILTV-01/2023, Vietnam/VNUA/ILTV-02/2023, Vietnam/VNUA/ILTV-03/2024, Vietnam/VNUA/ILTV-04/2024, Vietnam/VNUA/ILTV-05/2025, Vietnam/VNUA/ILTV-06/2025, Vietnam/VNUA/ILTV-07/2025, and Vietnam/VNUA/ILTV-08/2025, with abbreviated names set as VNUA-ILTV-01, VNUA-ILTV-02, VNUA-ILTV-03, VNUA-ILTV-04, VNUA-ILTV-05, VNUA-ILTV-06, and VNUA-ILTV-07, and VNUA-ILTV-08 (Table 1).

### 2.2. Characterization of gB, gJ, gG, and ICP4 Genes

Of the eight current ILTV strains, the present study obtained partial sequences for four genes (*gB*, *gJ*, *gG*, *ICP4*), with nucleotide identities of 99.74–100%, 99.63–100%, 99.66–100%, and 99.34–100%, respectively (Table 2(A–D)). Based on these partial *ICP4* gene sequences, the eight ILTV strains displayed maximum genetic divergences of 0.01, 0.01, 0.005, and 0.006 from the virulent ILTV, tissue culture origin (TCO) vaccine, attenuated vaccine, and chicken embryo origin (CEO) vaccine strains, respectively (Table 3).

Analysis of the amino acid (aa) sequence spanning residues 613–743 of the gB protein from the eight strains identified in the present study with reference sequences in GenBank showed variation at residue 644. Most strains contained threonine (T) at this position, whereas strains VNUA-ILTV-05 to VNUA-ILTV-08 contained isoleucine (I). Notably, all field ILTV strains had substitution of valine (V) with alanine (A) at residue 740 of the gB protein. In addition, there was a nucleotide polymorphism at position 1931 of the *gB* gene (Table 4). Specifically, strains VNUA-ILTV-01 to VNUA-ILTV-04 contained cytosine (C) at this position, whereas thymine (T) was the substitute in the other ILTV strains obtained in the present study.

Regarding the gJ protein, the aa sequence remained highly conserved across the ILTV strains analyzed in the present study. Nevertheless, the VNUA-ILTV-05 strain contained two aa substitutions (S357F and T359P) relative to the reference strains. Similar to the gB protein, the gG protein aa sequences of the eight ILTV strains were highly conserved. The only detected variation was a glycine (G)-to-alanine substitution at residue 220 (G220A), which was present consistently in strains VNUA-ILTV-03, VNUA-ILTV-04, and VNUA-ILTV-05 (Table 4).

Analysis of fragment 1 (aa positions 86–281) of the ICP4 protein showed only one aa polymorphism at residue 86. In detail, five strains retained asparagine (N) at this position, whereas strains VNUA-ILTV-01, VNUA-ILTV-03, and VNUA-ILTV-04 carried aspartic acid (D) (Table 4).

The number of base substitutions per site was calculated by averaging the overall sequence pairs between different groups. The analysis involved 23 nucleotide sequences. Codon positions consisted of 1st + 2nd + 3rd + non-coding. All positions contained gaps, with missing data eliminated. There were a total of 592 positions in the final data. Evolutionary analyses were conducted using MEGA 6.0 software.

### 2.3. Phylogenetic Characterization of gB, gJ, gG, and ICP4 Genes

**Figure 2:**
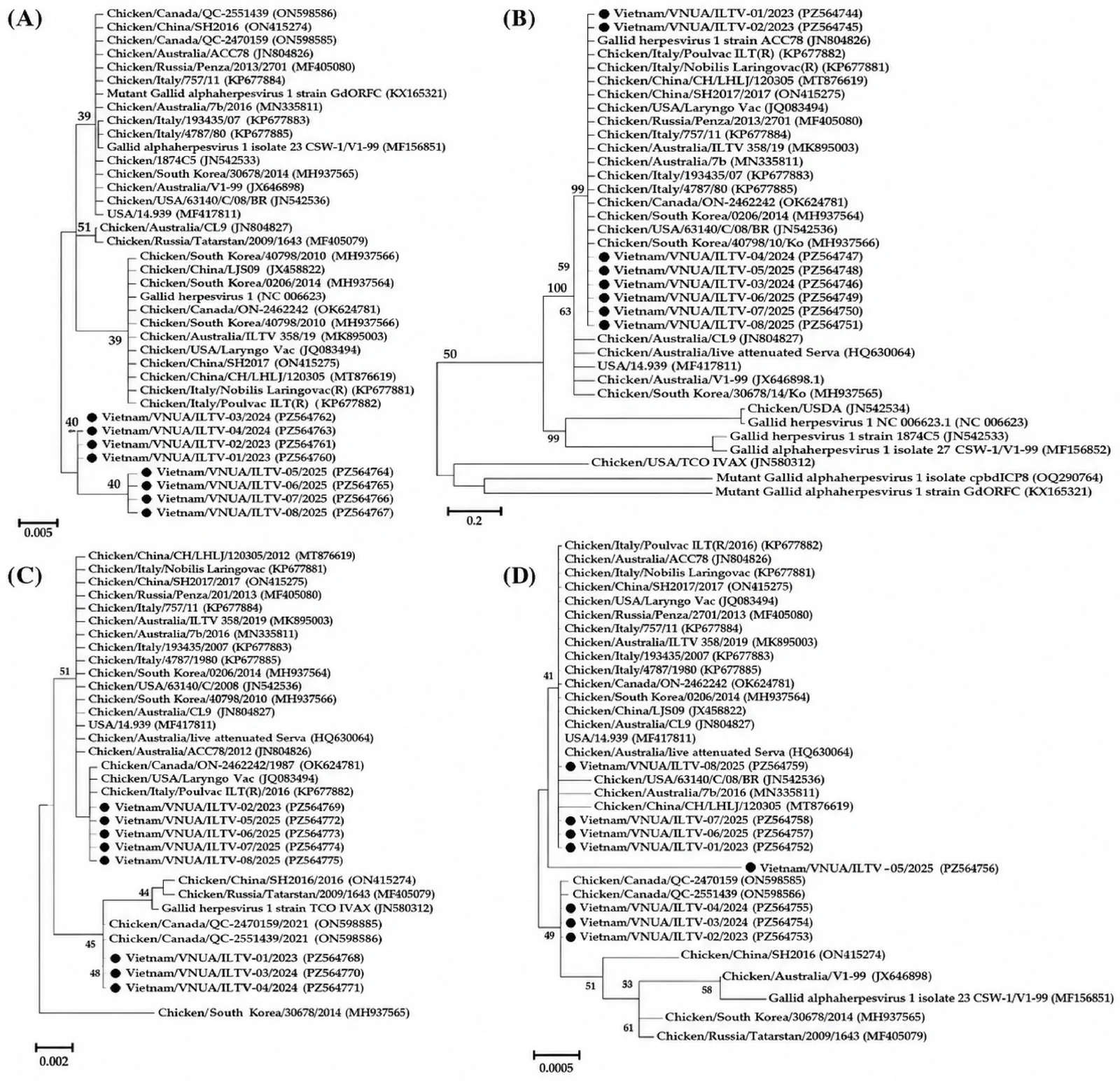
Phylogenetic tree of partial sequences of (**A**) *gB*, (**B**) *gG*, (**C**) *ICP4*, and (**D**) *gJ* genes of current Vietnamese ILTV strains compared to sequences of other ILTVs obtained from GenBank database. The tree was constructed using the maximum likelihood method (1000 bootstrap replicates) and MEGA6 software. Bootstrap values are shown at nodes. Sequences determined in the present study are highlighted with black circles.

The phylogenetic analyses of the eight representative ILTV strains using the four genes (*gB*, *gJ*, *gG*, and *ICP4* genes) revealed that all strains belonged to two distinct lineages, except for the *gJ* gene (Figure 2). In particular, the phylogenetic tree of the partial gB gene sequence indicated that four obtained ILTV strains (VNUA-ILTV-01 to VNUA-ILTV-04) were clustered together within a single group. In contrast, the remaining VNUA-ILTV strains formed a separate lineage. Notably, both lineages were differentiated clearly from the reference strains available in the GenBank database (Figure 2A). Similar to the *gB* phylogenetic analysis, the five identified ILTV strains (VNUA-ILTV-04 to VNUA-ILTV-08) belonged to a group with the live-attenuated Serva vaccine strain, whereas the remaining ILTV in the study were classified in one cluster with other ILTV vaccine strains (Poulvac ILT (Zoetis UK Limited, Surrey, UK), Nobilis Laringovac (MSD Animal Health, Cario, Egypt), USA Larynogo Vac (Zoetis US, New Jersey, US)) based on the phylogenetic tree of the *gG* gene (Figure 2B). Regarding the *ICP4* gene, the five ILTV strains (VNUA-ILTV-02, VNUA-ILTV-05 to VNUA-ILTV-08) belonged to a group containing the USA Laryno Vac, Poulvac-ILT, and ON-2462242/1987 strains. In addition, VNUA-ILTV-01, VNUA-ILTV-03, and VNUA-ILTV-04 were clustered in a distinct branch, based on the phylogenetic tree of the *ICP4* gene (Figure 2C).

The phylogenetic tree constructed using the partial *gJ* gene sequences indicated the presence of three distinct subgroups among the obtained ILTV strains. Specifically, VNUA-ILTV-02 to VNUA-ILTV-04 were clustered together and were closely related to an ILTV strain reported in Canada. Furthermore, the VNUA-ILTV-01 and VNUA-ILTV-06 to VNUA-ILTV-08 strains formed a separate subgroup that shared close genetic relatedness to vaccine strains and field isolates from Australia, China, and South Korea. Strain VNUA-ILTV-05 was assigned to an independent subgroup; however, this subgroup remained genetically closer to the strains VNUA-ILTV-01 and VNUA-ILTV-06 to VNUA-ILTV-08 than to the Canadian-associated lineage (Figure 2D).

## 3. Discussion

Although ILTV is considered to produce lower mortality rates than the highly pathogenic avian influenza virus (AIV) and the Newcastle disease virus (NDV), ILTV continues to be an economically important poultry pathogen. ILTV outbreaks can negatively affect poultry production and lead to substantial economic losses worldwide [12]. Before the present study, there had been only limited scientific reports documenting the occurrence or surveillance of ILTV in poultry production in Vietnam [11]. The present investigation has provided evidence of ILTV circulation in Vietnamese poultry farms, with PCR analysis targeting the *TK* gene detecting the virus genome in 8 of 14 farms examined, all located in Northern Vietnam. Other studies have reported that ILTV infection is characterized by clinical signs such as tracheal rales, severe tracheitis, respiratory distress, bloody tracheal exudate, reduced mortality, infraorbital sinus swelling, and conjunctival inflammation [7,8]. Gross pathological lesions observed at necropsy consisted of blood expectoration, blood clots in the tracheal lumen, hemorrhagic changes in the trachea and larynx, and congestion with edema of the suborbital sinus [8,13]. Similar clinical manifestations were observed in the chicken samples from ILT-suspected chicken flocks in the present study. However, despite the observation of clinical signs and post-mortem lesions, these findings are inadequate for diagnosing ILTV, largely due to the similarity with the respiratory signs induced by other economically important avian viruses, such as NDV, AIV, and infectious bronchitis virus. Consequently, confirmatory laboratory testing is essential for reliable identification of ILTV.

In the present study, the ILTV genome in the field samples was identified using PCR with a primer set to amplify the *TK* gene. Eight out of fourteen broiler farms suspected of having ILTV (57.14%) in the present study were positive for ILTV based on PCR in three provinces (TN, BN, and HN) in Northern Vietnam during 2023–2025. The partial sequences of four genes (*gJ*, *gB*, *gG*, and *ICP4*) were analyzed phylogenetically to characterize the genetic evolution of the ILTV strains currently circulating in Vietnam. Among the genomic targets used for this purpose, the *ICP4* gene plays a critical role because it regulates viral gene expression during the early phase of infection [3]. The *ICP4* gene has distinct sequence differences between the wild-type and vaccine strains [14]. Consistent with other reports, the phylogenetic tree based on the *ICP4* gene was divided into two major clusters, namely the TCO and CEO vaccines and the vaccine-like strains [15,16]. Among the isolates characterized in the present study, five ILTV strains were clustered within the vaccine-associated group and had the highest genetic similarity to CEO vaccine strains, particularly the USA Laryno Vac and Poulvac-ILT strains. This phylogenetic pattern is consistent with other published evidence suggesting that CEO vaccine-like or closely related ILTV strains have been associated with ILT outbreaks in poultry populations worldwide [17,18,19]. Nevertheless, because the vaccine strains used locally were not directly sequenced and compared with the isolates in the present study, these findings should not be interpreted as definitive evidence of vaccine derivation.

The gB protein, derived from the *UL27* gene, is an essential component of ILTV and is essential for viral binding to susceptible cells and subsequent viral internalization [20]. The present study identified the nucleotide variation at site 1931 of the *gB* gene among the strains examined, including both virulent and vaccine strains (TCO and CEO strains). In addition, another study demonstrated that most field strains had cytosine at this position, whereas most vaccine strains contained thymine [21]. These findings support the use of the nucleotide substitution at position 1931 in the *gB* gene as a potential genetic marker for differentiating field and vaccine-associated ILTV strains [21,22]. The codon composition at site 1931 in the *gB* gene indicated that the identified ILTV isolates showed sequence identity to vaccine strains and several field isolates.

In addition, analysis of the *gG* gene sequence has been used widely for the genetic characterization of ILTV isolates [23]. The sequence analysis in the present study identified a novel aa substitution (G to A) at position 220 in the gG protein of three field ILTV isolates in Vietnam. To the best of our knowledge, this was the first report of this mutation in either field or vaccine-related ILTV strains. Therefore, this supports undertaking additional surveillance and genetic characterization of ILTV strains presenting in the various regions of Vietnam to determine the distribution and epidemiological importance of this variant. Furthermore, ILTV gG is known to be an important virulence factor due to it linking with chemokines and suppressing leukocyte chemotaxis [24,25]. However, the functional effects of the identified mutations were not evaluated using bioinformatics-based structural of functional prediction tools. Therefore, further studies are essential to assess whether this aa substitution affects the biological properties and pathogenic potential of ILTV.

Phylogenetic and molecular analyses of the *gJ*, *gB*, *gG*, and *ICP4* genes from the ILTV strains isolated during 2023–2025 revealed that the outbreaks in commercial broiler flocks in Vietnam were associated with vaccine-related ILTV strains. Notably, none of the affected flocks included in the present study had been vaccinated against ILTV. In Vietnam, ILTV vaccination is practiced primarily in layer flocks, while, currently, chicken flocks are vaccinated mainly using CEO vaccines. It is considered a potential source of vaccine-like viruses. It has been suggested from other studies that the CEO and TCO vaccine viruses can increase virulence following bird-to-bird transmission, resulting in severe outbreaks among susceptible chickens [15,26]. In addition to vaccine-related factors, environmental transmission may contribute to ILTV spread. In particular, wind-borne dissemination of ILTV has been reported as an important epidemiological factor and may have facilitated virus transmission between poultry flocks [27,28]. In addition, the relatively small number of sampled farms and ILTV-positive isolates is a limitation of this study. This may not fully represent the genetic diversity of ILTV circulating in Northern Vietnam. Therefore, further studies involving larger-scale sampling are warranted to better characterize ILTV genetic diversity and transmission dynamics in the region.

## 4. Materials and Methods

### 4.1. Ethics Statement

All animal-related procedures (including handling and sample collection) were undertaken in strict compliance with the Ethical Guidelines of the Vietnam National University of Agriculture (CARE-2023/15 approval, 1 May 2023). The study protocol was approved by the University’s Committee on Animal Research and Ethics. Prior to sampling, written informed consent was obtained from all farm owners after providing a comprehensive explanation of the study objectives and sampling procedures. All procedures was performed in compliance with internationally recognized animal welfare standards to minimize animal stress and discomfort.

### 4.2. Sample Collection

Samples were collected from 14 commercial broiler farms in Thainguyen (TN), Bacninh (BN), and Hanoi (HN) cities/provinces in Northern Vietnam during 2023–2025. None of these flocks had been vaccinated against ILTV. The farm sites were located in TN, BN, and HN, with approximate distances of 81 km between TN and HN, 70 km between TN and BN, and 46 km between BN and HN. Five sick or dead chickens from each farm were sent for further analysis to the Department of Microbiology and Infectious Diseases, the Faculty of Veterinary Medicine, Vietnam National University of Agriculture. Pooled trachea swabs were collected in sterile tubes. A 10% (*w*/*v*) sample homogenate was prepared in phosphate-buffered saline supplemented with gentamicin (10 mg/mL).

### 4.3. DNA Extraction and PCR for ILTV Genome Detection

DNA was extracted from the homogenate sample using Viral Gene-spin™ Viral DNA/RNA Extraction Kit (iNtRON Biotechnology; Seoul, Republic of Korea), according to the manufacturer’s instructions.

The primer sets ILTV-F1 and ILTV-R1 were applied to amplify a 647 bp fragment of the partial thymidine kinase (*TK*) gene of ILTV (Table 5), as described elsewhere [29]. Subsequently, four additional primer sets were used for sequencing of the partial *ICP4*, *gB*, *gG*, and *gJ* genes (Table 5), as described elsewhere [30,31]. The thermal conditions as a preliminary denaturation step were 94 °C for 2 min, followed by 35 cycles at 94 °C for 30 s, 55 °C for 30 s, and 72 °C for 30 s, and a final extension step at 72 °C for 10 min. The PCR product was separated on 1.2% agarose gel and examined under UV light.

### 4.4. Nucleotide Sequencing and Phylogenetic Analysis

The PCR products were purified using GeneClean^®^ II Kits (MP Biomedicals; Santa Ana, CA, USA). Sequencing of the *ICP4*, *gB*, *gG*, and *gJ* genes was carried out by 1st BASE, Malaysia.

The sequence data were processed using the GENETYX ver. 10 software (GENETYX Corp.; Tokyo, Japan) and compared with reference sequences available using the BLAST algorithm (https://blast.ncbi.nlm.nih.gov/, accessed on 15 August 2026). Predicted amino acid (aa) sequences were generated using the Clustal W algorithm of BioEdit software version 7.2 [32,33]. Evolutionary distances were estimated from aligned sequences using the Kimura 2-parameter model. Phylogenetic trees were constructed using the maximum likelihood method in MEGA 6.0 software, with 1000 bootstrap replications [34].

### 4.5. Accession Numbers of Nucleotide Sequence

The nucleotide sequences generated in the present study were deposited in the GenBank database under accession numbers PZ564744–PZ564775.

## 5. Conclusions

The present study documented ILTV-positive field samples collected during 2023–2025 from unvaccinated commercial broiler farms with ILTV in Northern Vietnam. Molecular analyses of the *gJ*, *gB*, *gG*, and *ICP4* genes indicated that these ILTVs were genetically similar to known vaccine strains. These data have enhanced understanding of the molecular characterization of ILTV circulating in Northern Vietnam, with the findings contributing to the foundation of further studies on the viral evolution and transmission dynamics of ILTV. In addition, full-genome sequencing and comparative genomic analysis will be required to determine their origin in the future.

## Figures and Tables

**Table 1:** Characteristics of Vietnamese ILTV strains investigated in the present study.

Virus Strain	Year	District	Farm Type	Flock Size	GenBank Accession No.
VNUA-ILTV-01	2023	BN	Semi-Grazing	1500	PZ564744, PZ564752, PZ564760, PZ564769
VNUA-ILTV-02	2023	TN	Semi-Grazing	2500	PZ564745, PZ564753, PZ564761, PZ564768
VNUA-ILTV-03	2024	BN	Semi-Grazing	6000	PZ564746, PZ564754, PZ564762, PZ564770
VNUA-ILTV-04	2024	HN	Semi-Grazing	3000	PZ564747, PZ564755, PZ564763, PZ564771
VNUA-ILTV-05	2025	BN	Semi-Grazing	3000	PZ564748, PZ564756, PZ564764, PZ564772
VNUA-ILTV-06	2025	HN	Semi-Grazing	5000	PZ564749, PZ564757, PZ564765, PZ564773
VNUA-ILTV-07	2025	HN	Semi-Grazing	1000	PZ564750, PZ564758, PZ564766, PZ564774
VNUA-ILTV-08	2025	HN	Semi-Grazing	500	PZ564751, PZ564759, PZ564767, PZ564775

**Table 2:** (**A**) Nucleotide sequence identities of partial *gB* gene of Vietnamese ILTV strains. (**B**) Nucleotide sequence identities of partial *gJ* gene of Vietnamese ILTV strains. (**C**) Nucleotide sequence identities of partial *gG* gene of Vietnamese ILTV strains. (**D**) Nucleotide sequence identities of partial *ICP4* gene of Vietnamese ILTV strains.

**(A)**									
**Number of Strains**	**Strain Name**	**Number of Strains/nt Identity (%)**							
		1	2	3	4	5	6	7	8
1	VNUA/ILTV-01	100							
2	VNUA/ILTV-02	100	100						
3	VNUA/ILTV-03	100	100	100					
4	VNUA/ILTV-04	100	100	100	100				
5	VNUA/ILTV-05	99.74	99.74	99.74	99.74	100			
6	VNUA/ILTV-06	99.74	99.74	99.74	99.74	100	100		
7	VNUA/ILTV-07	99.74	99.74	99.74	99.74	100	100	100	
8	VNUA/ILTV-08	99.74	99.74	99.74	99.74	100	100	100	100
**(B)**									
**Number of Strains**	**Strain Name**	**Number of Strains/nt Identity (%)**							
		1	2	3	4	5	6	7	8
1	VNUA/ILTV-01	100							
2	VNUA/ILTV-02	99.94	100						
3	VNUA/ILTV-03	99.94	100	100					
4	VNUA/ILTV-04	99.94	100	100	100				
5	VNUA/ILTV-05	99.70	99.63	99.63	99.63	100			
6	VNUA/ILTV-06	100	99.94	99.94	99.94	99.70	100		
7	VNUA/ILTV-07	100	99.94	99.94	99.94	99.70	100	100	
8	VNUA/ILTV-08	100	99.94	99.94	99.94	99.70	100	100	100
**(C)**									
**Number of Strains**	**Strain Name**	**Number of Strains/nt Identity (%)**							
		1	2	3	4	5	6	7	8
1	VNUA/ILTV-01	100							
2	VNUA/ILTV-02	100	100						
3	VNUA/ILTV-03	99.66	99.66	100					
4	VNUA/ILTV-04	99.66	99.66	100	100				
5	VNUA/ILTV-05	99.66	99.66	100	100	100			
6	VNUA/ILTV-06	99.83	99.83	99.83	99.83	99.83	100		
7	VNUA/ILTV-07	99.83	99.83	99.83	99.83	99.83	100	100	
8	VNUA/ILTV-08	99.83	99.83	99.83	99.83	99.83	100	100	100
**(D)**									
**Number of Strains**	**Strain Name**	**Number of Strains/nt Identity (%)**							
		1	2	3	4	5	6	7	8
1	VNUA/ILTV-01	100							
2	VNUA/ILTV-02	99.34	100						
3	VNUA/ILTV-03	100	99.34	100					
4	VNUA/ILTV-04	100	99.34	100	100				
5	VNUA/ILTV-05	99.34	100	99.34	99.34	100			
6	VNUA/ILTV-06	99.34	100	99.34	99.34	100	100		
7	VNUA/ILTV-07	99.34	100	99.34	99.34	100	100	100	
8	VNUA/ILTV-08	99.34	100	99.34	99.34	100	100	100	100

**Table 3:** Comparative evolutionary distances among current Vietnamese ILTV and vaccine strains based on *ICP4* gene sequence.

Number of Strains	Strain Name	Virulent ILTV ^a^	TCO Vaccine ^b^	Attenuated Vaccine ^c^	CEO Vaccine ^d^
1	VNUA/ILTV-01	0–0.01	0.01	0.001	0–0.001
2	VNUA/ILTV-02	0.005–0.01	0.003	0.005	0.005–0.006
3	VNUA/ILTV-03	0.005–0.01	0.003	0.005	0.005–0.006
4	VNUA/ILTV-04	0.005–0.01	0.003	0.005	0.005–0.006
5	VNUA/ILTV-05	0–0.01	0.01	0.001	0–0.001
6	VNUA/ILTV-06	0–0.01	0.01	0.001	0–0.001
7	VNUA/ILTV-07	0–0.01	0.01	0.001	0–0.001
8	VNUA/ILTV-08	0–0.01	0.01	0.001	0–0.001

^a^: USA/63140/C/2008 strain (JN542536), Republic of South Korea/40798/2010 strain (MH937566), Republic of South Korea/30678/2014 strain (MH937565), China/CH/LHLJ/120305/2012 strain (MT876619), China/SH2017/2017 strain (ON415275), Canada/QC-2470159/2021 strain (ON598585), Canada/QC-2551439/2021 strain (ON598586), China/SH2016/2016 strain (ON152274). ^b^: TCO-IVAX strain (JN580312). ^c^: Live-attenuated Serva strain (HQ630064), Nobilis strain (KP677881). ^d^: Italy/4787/1980 strain (KP677885), Italy/193435/2007 strain (KP677883), LT Blent strain (JQ083493), USA/Laryngo vac strain (JQ083494), Poulvac strain (KP677882).

**Table 4:** Amino acid substitutions of representative gB, gJ, gG, and ICP4 proteins from other ILTV strains.

Strain Name	gB Protein (613–743)		gJ Protein (152–359)		gG Protein (34–229)	ICP4 Protein (86–281)
	644	740	357	359	220	86
Consensus ^a^	T/I	V	S	T/P	G	N/D
VNUA-ILTV-01	T	A	**.**	T	**.**	D
VNUA-ILTV-02	T	A	**.**	T	**.**	N
VNUA-ILTV-03	T	A	**.**	T	A	D
VNUA-ILTV-04	T	A	**.**	T	A	D
VNUA-ILTV-05	I	A	F	P	A	N
VNUA-ILTV-06	I	A	**.**	T	**.**	N
VNUA-ILTV-07	I	A	**.**	T	**.**	N
VNUA-ILTV-08	I	A	**.**	T	**.**	N
TCO and CEO vaccine ^c^	.^b^	.	.	.	.	.
Virulent strains ^d^	.	.	.	.	.	.

^a^: Consensus amino acid sequence derived from 57 ILTV strains from GenBank (PQ476026, MF417808–MF417811, PX492157, PQ497631, PQ476027, MK894996–MK895003, PX522223, PX496590, OR906269, NC_075683, NC_006623, JN596962, JN596963, JX646898, JX646899, JN804826, HQ630064, OL354140, OK646550, OK573459, KX165320, KX165321, PZ112168, PX619776, ON598585, ON598586, ON415274, JN808826, MF405080, KP677881–KP677885, MN335811, MF156851, JN542533, MH937564–MH937566, JN804827, MF405079, JX458822, OK624781, JQ083494, ON415275, MT876619). ^b^: Same as consensus amino acid sequence; ^c^: TCO-IVAX strain (JN580312), Italy/4787/1980 strain (KP677885), Italy/193435/2007 strain (KP677883), LT Blent strain (JQ083493), USA/Laryngo vac strain (JQ083494), Poulvac strain (KP677882), Nobilis strain (KP677881); ^d^: USA/63140/C/2008 strain (JN542536), South Korea/40798/2010 strain (MH937566), South Korea/30678/2014 strain (MH937565), China/CH/LHLJ/120305/2012 strain (MT876619), China/SH2017/2017 strain (ON415275), Canada/QC-2470159/2021 (ON598585), Canada/QC-2551439/2021 strain (ON598586), and China/SH2016/2016 strain (ON152274).

**Table 5:** Information on primer sets applied in present study.

Primer Name	Sequence (5′–3′) of Primer	Amplicon Length (bp)	Reference
TK-F	ACG ATG ACT CCG ACT TTC	647	[29]
TK-R	CGT TGG AGG TAG GTG GTA		
gB-F	CAA GGG CGG AAT TTG ATA GA	440	[31]
gB-R	AAT GAG GCG ATG CCA GAT GC		
gG-F	TTG TGC GCG TCT GTA TTA GG	612	[30]
gG-R	CTC CAT AGG ACC GTC GAG TT		
gJ-F	GTT AAC GCC TCT CTG GAA CG	667	[30]
gJ-R	TCG GGG AAG TAC CTG TAT CG		
ICP4-F	ACT GAT AGC TTT TCG TAC AGC ACG	688	[30]
ICP4-R	CAT CGG GAC ATT CTC CAG GTA GCA		

## Data Availability

The original data supporting the findings of this study are provided in the article. Further inquiries can be addressed to the corresponding author.
